# Enhancement of optical levitation with hyperbolic metamaterials

**DOI:** 10.1038/s41598-024-51284-4

**Published:** 2024-01-19

**Authors:** Ufuk Paralı, Kadir Üstün, İbrahim Halil Giden

**Affiliations:** 1 ASELSAN Inc., Mehmet Akif Ersoy Mah. İstiklal Marşı Cad. No:16, 06200 Yenimahalle-Ankara, Turkey; 2 Department of Electrical and Electronics Engineering, Faculty of Engineering, Gazi University, 06570 Ankara, Turkey

**Keywords:** Optical materials and structures, Micro-optics, Metamaterials, Optical manipulation and tweezers

## Abstract

The tightly focused laser beam in an optical trap has become a useful tool for many recent research areas. The momentum change in the photon-stream path of incident laser beam induces radiation force that enables trapping and manipulating mesoscopic micron-sized objects. In this study, we report the first analytical demonstration of optical trapping and levitation with radiation pressure on a transparent micron-sized spherical object made of hyperbolic metamaterial (HMM). The optical radial and axial forces acting on dielectric and HMM spherical particles are calculated using ray-optics approximation, assuming an optical levitation trapping setup. We compared the net force acting on the two objects, finding that the net radiation force exerted towards HMM particle is enhanced in the axial direction: The optical force enhancement in the HMM particle is more than ~ 8 times stronger compared to the induced force on the conventional dielectric particle with the corresponding material parameters. Besides, a better performance in the radial stabilization is observed for the HMM particle in comparison with the dielectric case, at which some oscillations and unstable saturation locations for the radial stabilization is monitored for *TEM*_00_ beam incidence. Furthermore, “zero-force” paths where radial stabilization of the HMM particle exists are also obtained for both *TEM*_00_ and $TEM_{01}^{*}$ laser beam incidences. Such phenomenon does not occur for particles of only dielectric and only metal material, which can be considered as another superiority of the proposed HMM particle.

## Introduction

It is an interesting technical challenge to trap and precisely control the movement of micron and sub-micron scale objects, without any mechanical contact. The first significant observation of optical levitation and trapping of particles by optical radiation forces has been reported by Nobel laureate Arthur Ashkin and his colleagues in the early 1970s ^1–8^. After Ashkin, the tightly focused laser beams have been a useful tool for scientific studies on the dynamics of single suspended micro-particles, molecules and biological cells in an optical levitation trap ^5–11^. On the other hand, there are not any experimental or numerical studies on the optical levitation and trapping of metamaterials or hyperbolic metamaterials even though there is a vast amount of literature on optical trapping of various micron-sized dielectric particles. To the best of our knowledge, this is the first time in the literature that; the numerical analysis and optical trapping performance comparison of transparent spherical micron-sized dielectric and hyperbolic metamaterial particles have been investigated.

**Figure 1:**
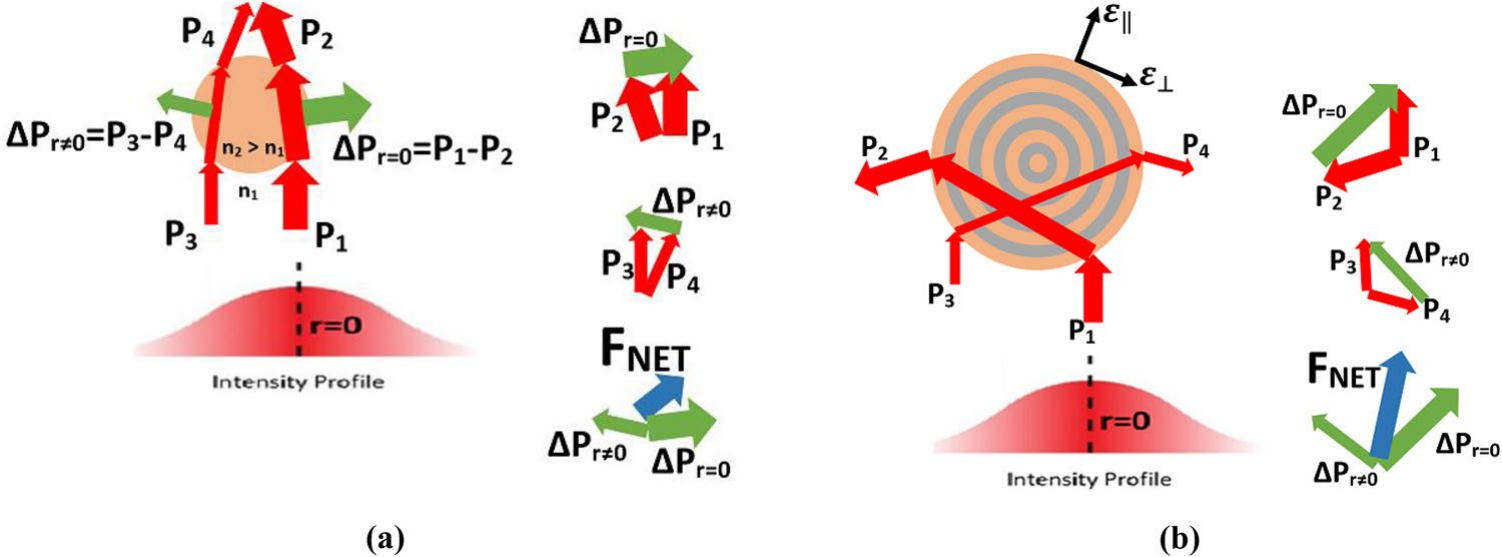
Conceptual representation of the momentum changes in the photon-stream path of incident laser beam that induces net radiation force on investigated micron-sized particles. (**a**) Momentum injection of the photon-stream path incident upon the lower side of a transparent micro-spherical dielectric particle with an arbitrarily given initial radial offset with respect to the center axis of the Gaussian beam. Here, $\Delta {\text{P}}_{r \ne 0} ,\Delta {\text{P}}_{r = 0}$, *n*_1_ and *n*_2_ denote the momentum change toward the center of laser beam (*r* = 0), momentum change outward of the laser beam (*r* ≠ 0), ambient refractive index and particle refractive index, respectively. (**b**) Momentum injection of the photon-stream path incident upon the lower side of a transparent micro-spherical Type-I HMM particle composed of Ge-AZO (Al-doped ZnO) dielectric/metal isocentric multi-shells from top to center. Beyond the reflected light off the surface, negative refraction inside the HMM sphere provides enhancement of the radiation pressure (axial force) through the propagation direction, which could be inferred from ray tracing schematic. Gradient force also arises through lateral direction due to negative refraction of light, which tends to pull the HMM particle to the center of the incident laser beam.

**Figure 2:**
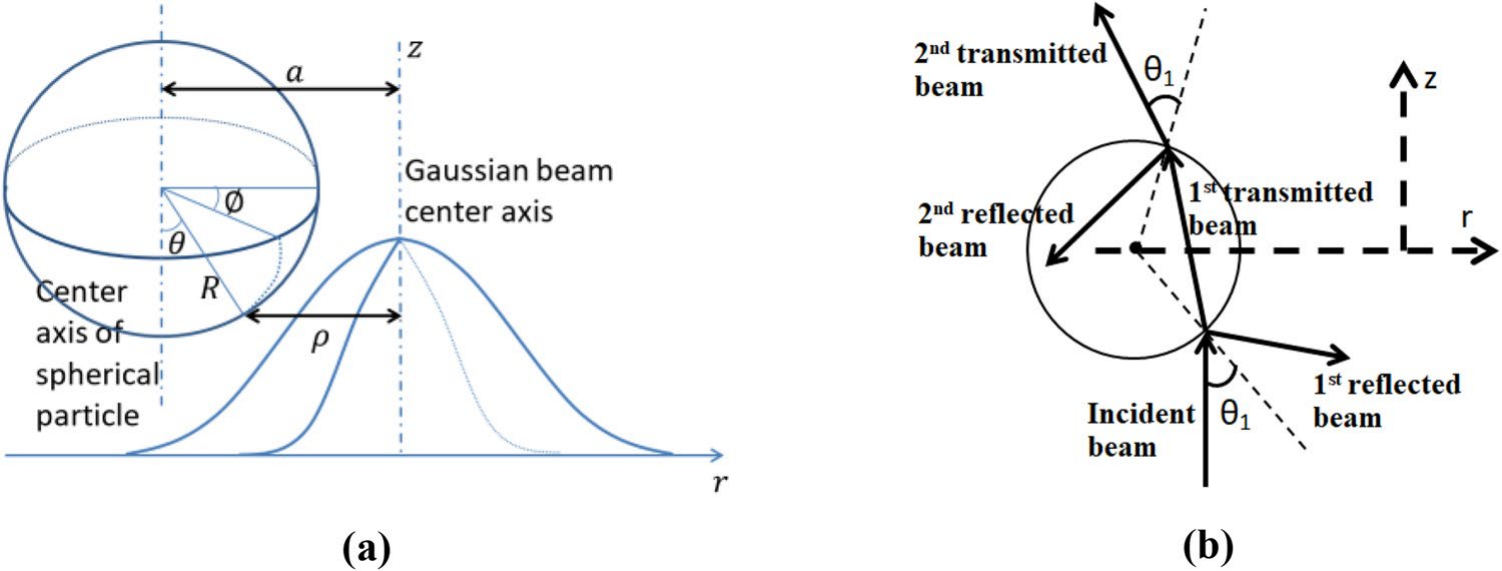
(**a**) 3-Dimensional view of a spherical particle under the illumination of a *TEM*_00_ Gaussian beam. Particle has an arbitrary initial radial offset with respect to the center axis of the beam. Here, $\emptyset$ is the polar angle, *θ* is the incident angle (and the elevation angle), *R* is the particle radius and |*a*| is the radial distance between the center axis of the beam and center axis of the sphere ^22^. (**b**) Reflected and transmitted ray-optics model of a dielectric transparent spherical particle under the exposure of a laser beam is schematically drawn. In this study, only optical forces due to the momentum change with respect to the 1st reflected & transmitted beams and 2nd reflected & transmitted beams are considered in both dielectric and hyperbolic metamaterial cases.

Optical metamaterials are artificial structures consisting of subwavelength-scale metal/dielectric components, allowing controllable manipulation of propagating light ^12^. The artificial materials has found a large number of optical applications such as high-resolution imaging ^13^, label-free sensing ^14^ and invisibility cloaking ^15^. Among different types of metamaterials such as chiral ^16^ and resonator-based metamaterials ^17^, hyperbolic metamaterials (HMMs) exhibit extraordinary optical characteristics with ease of fabrication, which attracted researchers’interests ^18^. HMMs are anisotropic media, possessing hyperbolic dispersion, by which optical negative refraction phenomenon becomes existent. The most intriguing optical characteristic of HMMs is to show negative electric response in one direction and positive electric response properties in another direction. That stems from the fact that HMMs can be modeled as uniaxial media having anisotropic permittivity tensors, formulated via the following relation:

1 $$\hat{\varepsilon } = \left[ {\begin{array}{*{20}c} {\varepsilon_{xx} } & 0 & 0 \\ 0 & {\varepsilon_{yy} } & 0 \\ 0 & 0 & {\varepsilon_{zz} } \\ \end{array} } \right],$$

where in-plane isotropic components are $\varepsilon_{xx} = \varepsilon_{yy} = \varepsilon_{{}}$, out-of-plane component is $\varepsilon_{zz} = \varepsilon_{ \bot }$, and the condition of $\varepsilon_{{}} \cdot \varepsilon_{ \bot } < 0$ should be satisfied to obtain hyperboloid isofrequency surfaces. “Hyperbolic” term is given to such anisotropic media due to topology of the created isofrequency surfaces, which is defined by the following relation:

2 $$\frac{{k_{x}^{2} + k_{y}^{2} }}{{\varepsilon_{ \bot } }} + \frac{{k_{z}^{2} }}{{\varepsilon_{{}} }} = k_{0}^{2} ,$$

where *k*_*x*_, *k*_*y*_, and *k*_*z*_ are the *x*, *y*, and *z* components of the wavevector of the propagating wave; $k_{0} = \omega /c$ is the free-space wavenumber, *ω* is the wave frequency and *c* is the speed of light. HMMs can be classified into two types in terms of the sign of permittivity tensors: In the case of $\varepsilon_{ \bot } < 0$ and $\varepsilon_{{}} > 0$, HMMs are termed as Type-I and isofrequency contours mimic two-fold hyperboloids. In the reverse case ($\varepsilon_{ \bot } > 0$ and $\varepsilon_{{}} < 0$), HMMs are called Type-II, having isofrequency contours with one-fold hyperboloid geometry. Compared to Type-II HMMs mostly exhibiting metallic characteristic, Type-I HMMs are relatively low-loss due to its inherent dielectric behavior ^19^. In this paper, we investigate the optical levitation and trapping behavior of micron-sized spherical dielectric particle (see Fig. 1a) and micron-sized spherical Type-I HMM particle composed of Ge/AZO (Al-doped ZnO) dielectric/metal isocentric multi-shells from top to center (see Fig. 1b). Since the studied particle with diameter 2*R* = 10 μm is larger than the operating SWIR/MWIR wavelengths, it can be modelled in Mie regime, at which geometric (ray) optics approach could be implemented to investigate the exerted optical forces on the mesoscopic object. Regarding ray-optics model of Ashkin’s approach, a net force occurs on the particle due to the change in the momentum of the photon-stream while passing through the transparent micro-spherical dielectric particle, as conceptually represented in Fig. 1a. This net force in the case of hyperbolic metamaterial is several multiple times greater than the dielectric material case, according to our analytical calculations. Negative light refraction at air-HMM boundary yields negative change of momentum, which should be compensated by an equal change in mechanical momentum of the particle in order to satisfy linear momentum conservation. The net force enhancement is schematically drawn in Fig. 1b. Here, the transverse direction is on the radial (*w.r.t.* beam center) axis and the longitudinal direction is on the propagation direction of the beam which is assumed to be on the z-dimension (as shown in Fig. 2a). Furthermore, a more consolidate radial position stabilization can be reached in the case of HMM particle instead of dielectric particle in the numerical optical setup.

In this study, ray-optics modelling for the axial and radial forces have been exactly adapted from ^20^ and the evolution of axial and radial dynamics are calculated utilizing Velocity Verlet integrator algorithm ^21^. For the numerical experiments, continuous wave (CW) *TEM*_00_ Gaussian beam (Eq. 3) and CW $TEM_{01}^{*} \left( {I_{{LG_{0}^{1} }} } \right)$ Laguerre-Gaussian beam (Eq. 4) are implemented and corresponding intensity profiles are defined, respectively, as ^22^;

3 $$I\left( {\rho ,z} \right) = \frac{2P}{{\pi W\left( z \right)^{2} }}exp\left[ {\frac{{ - 2\rho^{2} }}{{W\left( z \right)^{2} }}} \right]$$

and

4 $$I_{{LG_{0}^{1} }} \left( {\rho ,z} \right) = \frac{4P}{{\pi W\left( z \right)^{4} }}\rho^{2} \exp \left[ {\frac{{ - 2\rho^{2} }}{{W\left( z \right)^{2} }}} \right].$$

Here, as shown in Fig. 2a, z is the vertical displacement and *ρ* is the radial displacement of a point on the surface of the spherical particle where $$\theta \in \left[ {0,{\raise0.7ex\hbox{$\pi $} \!\mathord{\left/ {\vphantom {\pi 2}}\right.\kern-0pt} \!\lower0.7ex\hbox{$2$}}} \right]$$ and $$\emptyset \in \left[ {0,2\pi } \right]$$. *P* is the total power of the laser beam and $$W\left( z \right) = w_{0} \left[ {1 + \left( {{\raise0.7ex\hbox{$z$} \!\mathord{\left/ {\vphantom {z {z_{0} }}}\right.\kern-0pt} \!\lower0.7ex\hbox{${z_{0} }$}}} \right)^{2} } \right]^{1/2}$$ is the beam width where *w*_0_ is the beam waist and *z*_0_ is the Rayleigh range defined as $$z_{0} = {\raise0.7ex\hbox{${\pi w_{0}^{2} }$} \!\mathord{\left/ {\vphantom {{\pi w_{0}^{2} } {\lambda_{0} }}}\right.\kern-0pt} \!\lower0.7ex\hbox{${\lambda_{0} }$}}$$. Here *λ*_0_ is the wavelength of the laser beam.

Figure 2b represents the geometric ray-optics model of the photon-stream path of the incident laser beam on a transparent spherical dielectric particle. While calculating the radiation force on the dielectric and hyperbolic metamaterial particles, we only consider the momentum changes due to the 1st reflected & transmitted beams and 2nd reflected & transmitted beams ^20,22^.

Anisotropic optical response of HMMs could be determined via effective medium theory (EMT) in homogenization regime that the operating wavelength is much larger than the unit cell of HMMs, i.e. $\lambda_{incid} \gg t_{m} + t_{d}$, in which {*t*_*m*_, *t*_*d*_} are corresponding metal and dielectric thicknesses ^23^. Defining the filling fraction of metal layers as $f_{m} = \frac{{t_{m} }}{{t_{m} + t_{d} }}$, effective uniaxial dielectric tensors could be analytically calculated via the following relations;

5 $$\varepsilon_{{}} = f_{m} \varepsilon_{m} + \left( {1 - f_{m} } \right)\varepsilon_{d} ,$$

6 $$\varepsilon_{ \bot } = \frac{{\varepsilon_{m} \varepsilon_{d} }}{{f_{m} \varepsilon_{d} + \left( {1 - f_{m} } \right)\varepsilon_{m} }},$$

where the relative permittivities of metal and dielectric layers are termed as *ε*_*m*_ and *ε*_*d*_, respectively.

In this study, multilayered HMMs are preferred rather than nanowire structures due to the ease of fabrication via advanced physical vapor deposition as well as chemical vapor deposition methods ^24^. Proposed HMM multilayered particle is composed of alternating layers of Ge (as dielectric layer) and AZO (Al:ZnO as metal layer). Thicknesses of metal-dielectric pairs {*t*_*m*_, *t*_*d*_} are adjusted according to the filling fraction of metal layer, *f*_*m*_. This kind of multi-shell microspheres with adjustable metal-dielectric thicknesses could be realized by wet chemical synthesizing methods ^25^. In our study, the metal filling ratio is set to be *f*_*m*_ = 0.365 and the thicknesses of Ge-AZO metal-dielectric pairs are $\left\{ {t_{m} ,t_{d} } \right\} = \left\{ {0.042\,\upmu {\text{m}},0.024\,\upmu {\text{m}}} \right\}$. In this case, the thickness parameters of the proposed HMM particle is much smaller than the studied short-wave infrared and mid-wave infrared wavelengths and hence, the studied metaparticle can be modeled as an effective medium described by anisotropic Maxwell–Garnett theory ^26^. In the literature, other HMM structure designs are considered such as periodic nanowires. However, multilayered HMMs are preferred rather than nanowire structures in the study since multilayered HMM particles are much more practical in terms of fabrication and application of HMM concept. Nanowire structures needs careful construction of vertical wires onto a small spherical surface. Please also note that, it is difficult to preserve the constant periodic distance between wires on a spherical geometry, because, as the height of the wires increase, the flaring distance between nanowires increase. Even if we somehow implement nanowires onto the spherical surface satisfying the metamaterial design requirements, the surface itself would be made of a homogeneous material other than an artificial metamaterial which necessitates modelling of inhomogeneous spheres composed of homogeneous material and metamaterial.

**Figure 3:**
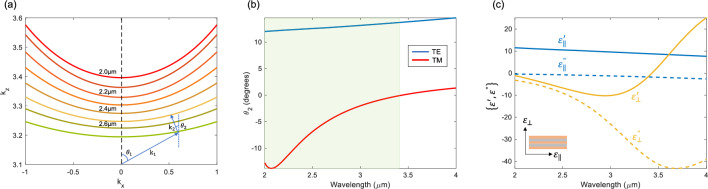
(**a**) Calculated isofrequency contours for varying operational wavelengths. Negative refraction phenomenon is schematically drawn as an inset. (**b**) Refraction angle spectra calculated for TE/TM polarizations with fixed incident angle *θ*_1_ = 45°. Negative refraction exists only when the incident beam is TM-polarized. The shaded region indicates the wavelength range of existing optical negative refraction. (**c**) Corresponding dielectric dispersion of the proposed Type-I HMM composed of Ge-AZO layers with *f*_*m*_ = 0.365.

The proposed system is investigated in SWIR/MWIR wavelengths, in which case Ge and AZO are good candidates for constructing the multilayered HMM particles. Permittivity values of Ge (*ε*_*d*_) and AZO (*ε*_*m*_) in IR wavelengths are provided from the experimental data in Refs. ^27,28^, respectively. Based on regarding {*ε*_*m*_, *ε*_*d*_} parameters with fixed *f*_*m*_ = 0.365, anisotropic dielectric tensor elements of the multilayered structure could be calculated via EMT relations (Eqs. 5–6) and corresponding wavevector components could be found via Eq. 2, as well. The calculated iso-frequency contours for different wavelengths are superimposed in Fig. 3a, exhibiting hyperbolic dispersion for TM polarization. The wavevectors are schematically drawn as inset in the figure to better visualize how incident wavevector *k*_1_ refracts at air-HMM interface when the horizontal component of refracted Poynting vector *S*_*x*,2_ is negative, in which circumstances optical negative refraction emerges in HMM particles. The refraction angle is computed for both polarizations according to the following discussions Section "Negative refraction phenomenon in hyperbolic metamaterials and the momentum transfer from optical beam to metaparticle". The results are given in Fig. 3b. It is clear from the figure that negative refraction (*θ*_2_ < 0°) only exists for TM polarization within $\lambda = 2.0\,\upmu {\text{m}} - 3.43\,\upmu {\text{m}}$ wavelengths. Anisotropic permittivity tensors defined as $\varepsilon_{xx} = \varepsilon_{yy} = \varepsilon_{{}} = \varepsilon_{{}}^{\prime } + i\varepsilon_{{}}^{\prime \prime }$ and $\varepsilon_{zz} = \varepsilon_{ \bot } = \varepsilon_{ \bot }^{\prime } + i\varepsilon_{ \bot }^{\prime \prime }$ are calculated and depicted in Fig. 3c. The out-of-plane dielectric tensor $\varepsilon_{ \bot }$ is negative within the operating wavelengths 2.0 μm–3.43 μm, whereas corresponding in-plane dielectric tensor *ε*_||_ remains positive, indicating that the proposed multilayered particle exhibits optical Type-I HMM characteristics. Within the specified wavelengths, the extreme anisotropy condition $\varepsilon_{ \bot } \cdot \varepsilon_{{}} < 0$ is satisfied, indicating the occurrence of negative refraction with the Ge-AZO multilayered structure.

## Negative refraction phenomenon in hyperbolic metamaterials and the momentum transfer from optical beam to metaparticle

In this section, our aim is to model the optical force that acts on the object and how the negative refraction boosts the momentum transfer. In the context of this paper, we mention electromagnetic plane wave propagation in the planar interface of a lossless double-positive isotropic medium and a uniaxial medium to show how the negative refraction occurs, using basic Electromagnetic Theory ^29^. We assume an isotropic medium for *z* < 0, a semi-infinite uniaxial medium for *z* > 0; and the optical axis is the *z*-axis. Here and for the rest of the section, subscripts “1” and “2” designates the isotropic medium and the uniaxial medium, respectively. We assume vacuum permeability for both sides $\mu_{1,2} = \mu_{0}$. The permittivity of the isotropic medium is *ε*_1_ and the refractive index of the medium can be found as $n_{1} = \sqrt {\varepsilon_{1} /\varepsilon_{0} }$ where *ε*_0_ is the vacuum permittivity. The permittivity tensor is given for uniaxial medium in Eq. 1. Without any loss of generality, we assume that the fields of the plane wave does not vary in *y*-direction, hence *y*-component of the wave vector is zero. Then, Eq. 2 simplifies to

7 $$\frac{{k_{x}^{2} }}{{\varepsilon_{ \bot } }} + \frac{{k_{z}^{2} }}{{\varepsilon_{{}} }} = k_{0}^{2} ,$$

As a matter of fact, for an isotropic medium, $\varepsilon_{{}} = \varepsilon_{ \bot }$. Here we analyze the behavior of the electromagnetic wave coming from an isotropic medium that encounters a boundary at *z* = 0 where half of the space *z* < 0 is filled by the isotropic medium and the other half *z* > 0 is filled with the uniaxial medium. Our purpose is to find reflected and transmitted power, and their propagation directions at the interface. Once we find these quantities, we can fit our findings to spherical interface, and we can find the momentum transfer to the sphere assuming theadius of curvature is large enough to be modeled as planar. In a planar interface, there are two main boundary conditions: Electric and Magnetic field components that are tangential to the interface are continuous. Therefore, the tangential component of the wave vector is also continuous. In our case, the continuous component of the wave vector is *k*_*x*_: $k_{x,1} = k_{x,2}$, and the *x*- and *y*-components of the electric field and the magnetic field are continuous at dielectric/metal interface. The incidence angle of the incoming wave is *θ*, hence the wave vector components in the isotropic medium are $k_{x,1} = n_{1} k_{o} \sin \theta_{1}$ and $k_{z,1} = n_{1} k_{o} \cos \theta_{1}$. As the *k*_*x*_ component is continuous in the interface, $k_{x,2} = n_{1} k_{o} \sin \theta_{1}$. Then, we can find the *z*-component of the wave vector in the uniaxial medium, using Eq. 7. Once we have found the wave vector components, we can deduce the refraction angle.

Proper definition of the propagation direction is indispensable to find out the correct refraction angle. Here we claim that, the propagation direction is the direction of the Poynting’s vector, the vector that show how the power propagates. Let $\vec{E}$ and $\vec{H}$ be electric and magnetic fields, respectively, in phasor form with $e^{j\omega t}$ temporal dependency. The Poynting’s vector is $\vec{S} = \vec{E} \times \vec{H}^{*} = S_{x} \vec{a}_{x} + S_{z} \vec{a}_{z}$ (without loss of generality we have *S*_*y*_ = 0, otherwise the plane wave fields would necessitate non-zero *k*_*y*_ values for isotropic and uniaxial medium contrary to our assumption *k*_*y*_ = 0). Also note that the boundary plane is at *z* = 0, therefore the *y*-component of the wave vector is tangential and hence continuous. As the wave vector of the incident wave has no *y*-component, neither do the reflected and transmitted waves). As we are interested in the time-average power propagation, $\vec{S}_{av} = \frac{1}{2}{\text{Re}} \left\{ {\vec{E} \times \vec{H}^{*} } \right\} = \frac{1}{2}{\text{Re}} \left\{ {S_{x} } \right\}\vec{a}_{x} + \frac{1}{2}{\text{Re}} \left\{ {S_{z} } \right\}\vec{a}_{z}$, because the fields are in phasor form and only the real parts give us time-average power propagation. As we find the directions of the power transfer, the angle of power propagation *θ* can be found using $\tan \theta = \frac{{{\text{Re}} \left\{ {S_{x} } \right\}}}{{{\text{Re}} \left\{ {S_{z} } \right\}}}$. In the lossless isotropic medium this relation corresponds to $\tan \theta_{1} = \frac{{k_{x,1} }}{{k_{z,1} }}$. However, the situation is a bit different for uniaxial medium. Without delving into the details, ${\text{Re}} \left\{ {S_{z} } \right\} > 0$ in the uniaxial medium, because the incident field is propagating in the + *z*-direction in the isotropic medium (for *z* < 0), the interface is at *z* = 0, and the electromagnetic wave can only couple to the + *z*-direction, otherwise this would contradict with the existence of the wave inside the uniaxial medium in *z* > 0. To attain negative values for the angle of power propagation in uniaxial medium (*θ*_2_ < 0) for positive values of the incidence angle *θ*_1_, we should have Re{*S*_*x*_} < 0 ($\tan \theta_{2} = \frac{{{\text{Re}} \left\{ {S_{x} } \right\}}}{{{\text{Re}} \left\{ {S_{Z} } \right\}}} < 0$, and Re{*S*_*z*_} > 0). If the incidence angle is positive, the transverse component of the wave vector is *k*_*x*_ is also positive. To reveal the wave behavior in the uniaxial medium, without loss of generality, we can decompose the Electromagnetic Plane Wave into two polarizations: Transverse Electric (TE) and Transverse Magnetic (TM) waves. The Electric (Magnetic) field has only *y*-component for TE(TM) wave. Then TE wave has {*E*_*y*_, *H*_*x*_, *H*_*z*_} components and TM wave has {*H*_*y*_, *E*_*x*_, *E*_*z*_} components. Using the Maxwell’s Equations, it can be found that:

8a $$S_{x} = \frac{{k_{x}^{*} }}{{\omega \mu_{0} }}\left| E \right|^{2}$$

8b $$S_{z} = \frac{{k_{z}^{*} }}{{\omega \mu_{0} }}\left| E \right|^{2}$$

For TE polarization. It can be noted that Re{*S*_*x*_} has the same direction with Re{*k*_*x*_}, and negative refraction is impossible. For TM polarization:

9a $$S_{x} = \frac{{k_{x} }}{{\omega \varepsilon_{ \bot } }}\left| H \right|^{2}$$

9b $$S_{z} = \frac{{k_{z} }}{{\omega \varepsilon_{{}} }}\left| H \right|^{2}$$

Then for $\varepsilon_{ \bot }$ with negative real part, the negative refraction can occur. Accordingly, Type I metamaterials can show negative refraction behavior for only TM waves, for non-magnetic case (*μ* = *μ*_0_). We computed the refraction angle for both TE and TM waves, using the formula:

10 $$\theta_{2} = \tan^{ - 1} \frac{{{\text{Re}} \left\{ {S_{x,2} } \right\}}}{{{\text{Re}} \left\{ {S_{z,2} } \right\}}}$$

After some detailed analysis, we found the refraction angle in the HMM medium as:

11a $$\theta_{2,TE} = \tan^{ - 1} \left( {\frac{{n_{1} k_{o} \sin \theta_{1} }}{{Real\left\{ {\sqrt {\left( {\frac{{\varepsilon_{||} }}{{\varepsilon_{o} }}} \right)k_{o}^{2} - n_{1}^{2} k_{o}^{2} \left( {\sin \theta_{1} } \right)^{2} } } \right\}}}} \right)$$

11b $$\theta_{2,TM} = \tan^{ - 1} \left( {\frac{{Real\left\{ {\varepsilon_{ \bot }^{*} } \right\}}}{{Real\left\{ {\varepsilon_{||}^{*} \sqrt {{\raise0.7ex\hbox{${\varepsilon_{||} }$} \!\mathord{\left/ {\vphantom {{\varepsilon_{||} } {\varepsilon_{o} }}}\right.\kern-0pt} \!\lower0.7ex\hbox{${\varepsilon_{o} }$}}\left( {1 - \frac{{n_{1}^{2} \left( {\sin \theta_{1} } \right)^{2} }}{{{\raise0.7ex\hbox{${\varepsilon_{ \bot } }$} \!\mathord{\left/ {\vphantom {{\varepsilon_{ \bot } } {\varepsilon_{o} }}}\right.\kern-0pt} \!\lower0.7ex\hbox{${\varepsilon_{o} }$}}}}} \right)} } \right\}}}\frac{{\left| {\varepsilon_{||} } \right|^{2} }}{{\left| {\varepsilon_{ \bot } } \right|^{2} }}n_{1} \sin \theta_{1} } \right)$$

for the two polarizations. We can also assign an effective refractive index that depends on the incidence angle using the Snell’s Law:

12 $$n_{2eff,TE,TM} \left( {\theta_{1} } \right) = \frac{{n_{1} \sin \theta_{1} }}{{\sin \theta_{TE,TM} }}$$

for each polarization.

It was previously mentioned that *k*_*x*_ component of the wave vector is determined by the incidence angle *θ*_1_ as $k_{x,1} = n_{1} k_{o} \sin \theta_{1}$. The other components can be found using the following formulae:

13a $$k_{z,1} = \sqrt {\varepsilon_{0} n_{1}^{2} k_{0}^{2} - k_{x}^{2} }$$

13b $$k_{z,2} = \sqrt {\varepsilon_{{}} \left( {k_{0}^{2} - \frac{{k_{x}^{2} }}{{\varepsilon_{ \bot } }}} \right)}$$

After finding the wave vector components *k*_*x*_ and *k*_*z*_, in order to find the optical force acting on the HMM object, we also need to find the ratio of the fields of the reflected and transmitted fields to the incident field. Using the continuity of the tangential field components, we find the power reflectance and transmittance coefficients as;

14a $$R_{TE} = \left| {\frac{{E_{TE}^{r} }}{{E_{TE}^{i} }}} \right|^{2}$$

14b $$R_{TM} = \left| {\frac{{H_{TM}^{r} }}{{H_{TM}^{i} }}} \right|^{2}$$

where

15a $$\frac{{E_{TE}^{r} }}{{E_{TE}^{i} }} = \frac{{k_{z,1} - k_{z,2} }}{{k_{z,1} + k_{z,2} }}$$

15b $$\frac{{H_{TM}^{r} }}{{H_{TM}^{i} }} = \frac{{\frac{{k_{z,1} }}{{\omega n^{2} \varepsilon_{0} }} - \frac{{k_{z,2} }}{{\varepsilon_{{}} }}}}{{\frac{{k_{z,1} }}{{\omega n^{2} \varepsilon_{0} }} + \frac{{k_{z,2} }}{{\varepsilon_{{}} }}}}$$

Thus, we have the reflectance coefficient for the two interfaces (air to HMM and HMM to air) for unpolarized light:

16 $$\left| {r_{1} } \right|^{2} = \frac{{R_{TE} + R_{TM} }}{2},\quad \left| {r_{2} } \right|^{2} = \left| {r_{1} } \right|^{2}$$

and the transmittance coefficient:

17 $$\left| {t_{1} } \right|^{2} = 1 - \left| {r_{1} } \right|^{2} ,\quad \left| {t_{2} } \right|^{2} = \left| {t_{1} } \right|^{2}$$

Here, subscript 1 corresponds to the first reflection and transmission that occur in the ambient medium and the particle and subscript 2 corresponds to second reflection/transmission, accordingly. However, as the negative refraction effect is to be more pronounced, in our calculations we assume that the incoming light is TM polarized, hence $\left| {r_{1} } \right|^{2} = \left| {r_{2} } \right|^{2} = R_{TM}$.

Finally, we can explicitly define the optical axial and radial forces due to the reflection and transmission in a transparent micro-spherical Type-I HMM particle. As aforementioned, the radiation force calculations are based on the analytical derivations in ^20^, which are for only transparent spherical dielectric particle. In this study, following radiation force equations are derived for transparent spherical Type-I HMM particle;

18 $$F_{1rz} = \mathop \smallint \limits_{0}^{\pi /2} \frac{\pi }{c}n_{1} \left[ {1 + Cos\left( {2\theta_{1} } \right)} \right]I\left( {\rho ,z} \right)\left| {r_{1} } \right|^{2} R^{2} Sin\left( {2\theta_{1} } \right)d\theta_{1}$$

19 $$F_{1tz} = \mathop \smallint \limits_{0}^{\pi /2} \frac{\pi }{c}\left[ {n_{1} - n_{2eff,TE,TM} Cos\left( {\theta_{1} - \theta_{2,TE,TM} } \right)} \right]I\left( {\rho ,z} \right)\left| {t_{1} } \right|^{2} R^{2} Sin\left( {2\theta_{1} } \right)d\theta_{1}$$

20 $$F_{2rz} = \mathop \smallint \limits_{0}^{\pi /2} \frac{\pi }{c}n_{2eff,TE,TM} \left[ {Cos\left( {\theta_{1} - \theta_{2,TE,TM} } \right) + Cos\left( {3\theta_{2,TE,TM} - \theta_{1} } \right)} \right]I\left( {\rho ,z} \right)\left| {t_{1} } \right|^{2} \left| {r_{1} } \right|^{2} R^{2} Sin\left( {2\theta_{1} } \right)d\theta_{1}$$

21 $$F_{2tz} = \mathop \smallint \limits_{0}^{\pi /2} \frac{\pi }{c}\left\{ {n_{2eff,TE,TM} Cos\left( {\theta_{1} - \theta_{2,TE,TM} } \right) - n_{1} Cos\left[ {2\left( {\theta_{1} - \theta_{2,TE,TM} } \right)} \right]} \right\}I\left( {\rho ,z} \right)\left| {t_{1} } \right|^{2} \left| {t_{1} } \right|^{2} R^{2} Sin\left( {2\theta_{1} } \right)d\theta_{1}$$

22 $$F_{1rr} = - \mathop \smallint \limits_{0}^{\pi /2} \mathop \smallint \limits_{0}^{2\pi } I\left( {\rho ,z} \right)\frac{{n_{1} }}{2c}Sin\left( {2\theta_{1} } \right)\left| {r_{1} } \right|^{2} R^{2} Cos\left( \phi \right)Sin\left( {2\theta_{1} } \right)d\phi d\theta_{1}$$

23 $$F_{1tr} = \mathop \smallint \limits_{0}^{\pi /2} \mathop \smallint \limits_{0}^{2\pi } I\left( {\rho ,z} \right)\frac{{n_{2eff,TE,TM} }}{2c}Sin\left( {\theta_{1} - \theta_{2,TE,TM} } \right)\left| {t_{1} } \right|^{2} R^{2} Cos\left( \phi \right)Sin\left( {2\theta_{1} } \right)d\phi d\theta_{1}$$

24 $$F_{2rr} = \mathop \smallint \limits_{0}^{\pi /2} \mathop \smallint \limits_{0}^{2\pi } I\left( {\rho ,z} \right)\frac{{n_{2eff,TE,TM} }}{2c}\left[ {Sin\left( {3\theta_{2,TE,TM} - \theta_{1} } \right) - Sin\left( {\theta_{1} - \theta_{2,TE,TM} } \right)} \right]\left| {t_{1} } \right|^{2} \left| {r_{1} } \right|^{2} R^{2} Cos\left( \phi \right)Sin\left( {2\theta_{1} } \right)d\phi d\theta_{1}$$

25 $$F_{2tr} = \mathop \smallint \limits_{0}^{\pi /2} \mathop \smallint \limits_{0}^{2\pi } \frac{{I\left( {\rho ,z} \right)}}{2c}\left\{ {n_{1} Sin\left[ {2\left( {\theta_{1} - \theta_{2,TE,TM} } \right)} \right] - n_{2eff,TE,TM} Sin\left[ {\left( {\theta_{1} - \theta_{2,TE,TM} } \right)} \right]} \right\}\left| {t_{1} } \right|^{2} \left| {t_{1} } \right|^{2} R^{2} Cos\left( \phi \right)Sin\left( {2\theta_{1} } \right)d\phi d\theta_{1}$$

Here, *F*_1*rz*_ and *F*_2*rz*_ are the axial forces due to the 1st and 2nd reflection, respectively. *F*_1*tz*_ and *F*_2*tz*_ are the axial forces due to the 1st and 2nd transmission, respectively. *F*_1*rr*_ and *F*_2*rr*_ are the radial forces due to the 1st and 2nd reflection, respectively. *F*_1*tr*_ and *F*_2*tr*_ are the radial forces due to the 1st and 2nd transmission, respectively. In the analytical calculations, we use the following values for the given parameters in Table 1.

**Table 1:** Parameters used in the simulations of optical trapping dynamics of transparent spherical particle.

Input parameters	Value
Initial radial offset (µm)—*r*_*i*_	3.0
Initial axial offset (mm)—*z*_*i*_	− 5.0
Initial axial velocity (ms^−1^)—v_i_	5.0
Type-I HMM and Dielectric Particle radius (µm)—*R*	5.0
Beam waist (µm)—*w*_0_	4.0
Laser power (mW)—*P*	200
Density of the Type-I HMM (Ge-AZO) particle (kg/m^3^)	5400
Density of the dielectric particle (kg/m^3^)	5320
Medium dynamic viscosity (kgm^−1^ s^−1^)	1.82 × 10^−6^
Laser wavelength (µm)—*λ*_0_	2.4
Simulation step size (s)—Δt	5 × 10^−4^
Ambient refractive index—*n*_1_	1.0
Type-I HMM in-plane permittivity *ε*_*xx*_ = *ε*_*yy*_	10.71 − j0.6631
Type-I HMM out-of-plane permittivity *ε*_*zz*_	− 5.523 − *j*7.371
Corresponding dielectric particle refractive index—*n*_2_	$\sqrt {10.71}$
Type-I HMM particle effective refractive index—*n*_2*eff*_	Calculated from derivations—see Section "Negative Refraction Phenomenon In Hyperbolic Metamaterials And The Momentum Transfer From Optical Beam To Metaparticle"

## Results and discussion

**Figure 4:**
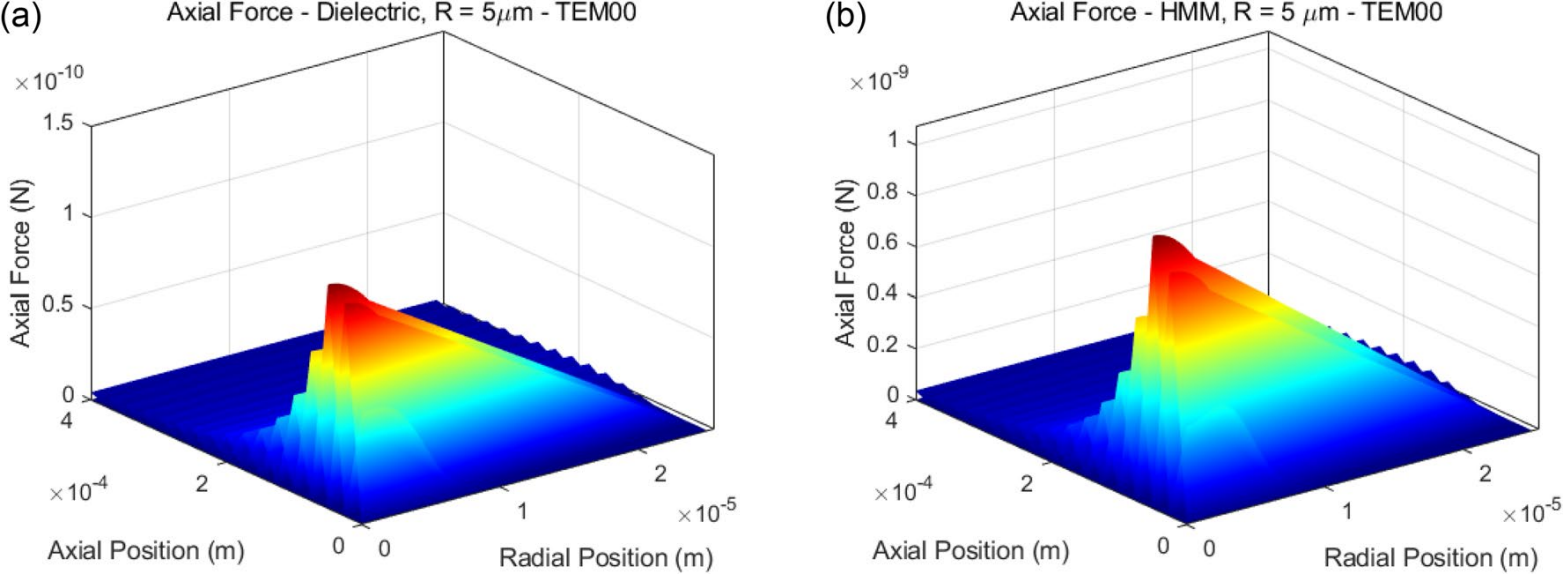
In this figure, only optical forces are considered. (**a**) Optical force in the axial direction on the transparent micro-spherical dielectric particle. (**b**) Optical force in the axial direction on the transparent micro-spherical Type-I HMM particle.

The axial and radial forces and their effect on the evolution of axial and radial dynamics are calculated utilizing Velocity Verlet integrator algorithm ^21^, based on the above-mentioned analytical derivations. Accordingly, for the ease of computation, the attenuation of propagating light through the HMM medium is ignored. The net optical forces assuming excitation with TM polarization (the polarization that negative refraction occurs) accumulated in the axial direction in the case of dielectric and HMM particles are calculated for *TEM*_00_ laser beam incidence, see Fig. 4. For the dielectric particle case, the peak value of the exerted optical force is obtained to be 1.232 × 10^−10^ N while the peak value is 1.072 × 10^−9^ N for the HMM particle, which is ~ 8.7 times greater than the conventional dielectric particle’s case.

In addition to the axial force profiles given for 2*R* = 10 μm, we also include the axial force profiles for some other radius choices, see Fig. S1 in the Supplementary Information. The maximum force values for a dielectric sphere and a HMM sphere as well as the optical force enhancement ratios $\frac{{max\left( {F_{axial,HMM} } \right)}}{{max\left( {F_{axial,Dielectric} } \right)}}$ are given in Table 2. It can be noted that, the force enhancement ratio stays nearly constant for varying diameters of the particles.

**Table 2:** Optical force enhancement ratio depending on varying particle diameters.

	Maximum axial force on dielectric particle (N)	Maximum axial force on HMM particle (N)	Force enhancement ratio
R = 5 µm	1.232 × 10^−10^	1.072 × 10^−9^	8.7
R = 7.5 µm	1.28 × 10^−10^	1.105 × 10^−9^	8.63
R = 10 µm	1.333 × 10^−10^	1.128 × 10^−9^	8.46
R = 20 µm	1.356 × 10^−10^	1.165 × 10^−9^	8.59

This kind of overwhelming optical force enhancement in the case of HMM particles could be explained by the change of photon momentum while entering inside the HMM sphere: Even though the speed of light as well as its wavelength is reduced by ratio of effective refractive index $${\raise0.7ex\hbox{$1$} \!\mathord{\left/ {\vphantom {1 {n_{eff} }}}\right.\kern-0pt} \!\lower0.7ex\hbox{${n_{eff} }$}}$$, the frequency of light ω is unchanged, meaning that the energy of photon remains constant while entering the HMM sphere. The light momentum inside a dielectric object is defined by the following Minkowski’s momentum relation:

26 $$p =n_{eff} \hbar\omega/c$$

If the photon enters the sphere (with effective refractive index *n*_*eff*_) from air (with refractive index *n*_1_ = 1) at normal incidence, the change of momentum is found to be such as the following relation:

27 $$\Delta p =\left( {n_{eff} - 1} \right)\hbar\omega/c$$

Considering that the laser pulse is normally incident with pulse duration *τ* and the total number of photon is count to be *N*, the radiation force acting at air-HMM interface is defined as the following expression ^30^:

28 $$F = {\raise0.7ex\hbox{${\left( {n_{eff} - 1} \right)\hbar\omega N}$} \!\mathord{\left/ {\vphantom {{\left( {n_{eff} - 1} \right)\hbar\omega N} {c\tau }}}\right.\kern-0pt} \!\lower0.7ex\hbox{${c\tau }$}} = {\raise0.7ex\hbox{${\left( {n_{eff} - 1} \right)P}$} \!\mathord{\left/ {\vphantom {{\left( {n_{eff} - 1} \right)P} c}}\right.\kern-0pt} \!\lower0.7ex\hbox{$c$}}$$

where *P* is the optical power of incident laser pulse.

**Figure 5:**
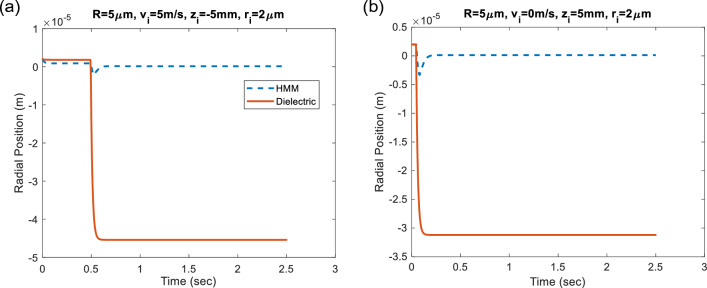
Radial stabilization performance of transparent micro-spherical Type-I HMM and dielectric particles (**a**) with initial velocity v_i_ and (**b**) of the particles at rest.

In case of negative refraction *n*_*eff*_ < 0, phase as well as group velocities of propagating light inside HMM particle are antiparallel, implying that phase and group wavefronts propagate in the opposite directions. In this situation, negative momentum change of photon exists while crossing the air-HMM boundary ^31^ and hence, the HMM sphere is subject to negative light pressure in the direction of incident laser pulse, causing the HMM particle to move upward direction, see Fig. 1b. The superiority of using HMM particle for enhanced optical levitation emerges when the incident beam enters the sphere: the light beam exposes to negative refraction as illustrated in Fig. 1b and reaches to HMM-air interface. Contrary to air-HMM interface case, positive momentum change of photon is gathered at HMM-air boundary and hence, the existing positive light pressure pulls again the HMM particle upward direction ^32^. The net accumulation of both existing radiation forces at air-HMM as well as HMM-air interfaces provides the resultant force enhancement (more than ~ 8 times stronger than conventional dielectric particle’s case) in such HMM particles. Figure 5a, b demonstrate the radial stabilization performance of two particles under the same *TEM*_00_ radiation pressure with and without initial velocity, respectively. The HMM particle has dramatically superior stabilization performance than the dielectric particle that oscillates and then, is pushed away from the beam axis of the applied optical radiation. On the other hand, HMM particle stabilizes radially after a short while near to the center of the laser beam axis, see Fig. 5. In order to ensure the inclusiveness of the analyses, we made further calculations for different input parameters. The axial offset and initial velocity parameters are fixed and the radial stabilization performance of dielectric/HMM particle with particle radii *R* = 5 μm is calculated based on varying radial offset, see Fig. S3 in the Supplementary Information.

In the preceding dynamic Velocity Verlet analyses, random Brownian motion of fluidic particles due to thermal effects has not been considered. Since thermal instabilites between the ambient medium and the HMM microparticle lead to non-equilibrium thermal fluctuations, the relaxation dynamics of the fluctuating HMM particle could be described by Langevin equation via the following relation ^33^:

29 $$F_{th} = \sqrt {2m{\Gamma }_{0} k_{B} T_{0} } \zeta \left( t \right).$$

**Figure 6:**
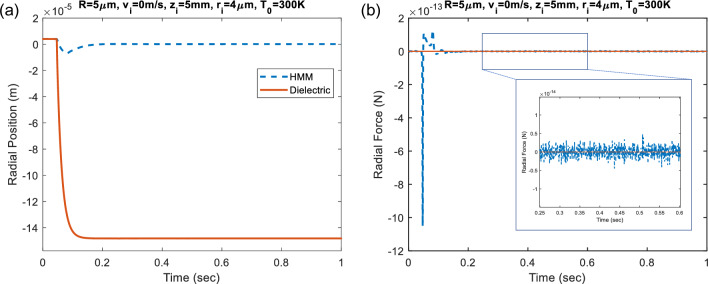
Time dependent (**a**) radial position and (**b**) radial force calculations under the non-equilibrium fluctuations.

In this equation, *m* is mass of the particle; Γ_0_ is the deterministic damping that depends on viscosity of the medium with the following formula, $\Gamma_{0} = 6\pi R\mu$, where μ is the viscousity of the medium. *k*_*B*_ is Boltzmann’s constant and *T*_0_ is the bath temperature, which is assumed to be the room temperature *T*_0_ = 300 K. The stochiastic function *ζ*(*t*) is white noise with delta correlation. Radial stabilization performance of both dielectric and HMM particles are explored while applying time-dependent external fluctuation force *F*_*th*_ for sufficiently long time. Corresponding radial time position as well as radial force calculations are represented in Fig. 6a, b, respectively. It is obvious from the calculations that although non-equilibrium fluctuations are observed at initial state, a balanced fluctuation is obtained with negligible oscillations at equilibrium state after sufficient period of time and the radial stabilization is still preserved in the case of HMM particle, see Fig. 6a. Negligible radial force oscillations are investigated, given as an inset in Fig. 6b, which implies the system relaxation from non-equilibrium towards transient steady states.

**Figure 7:**
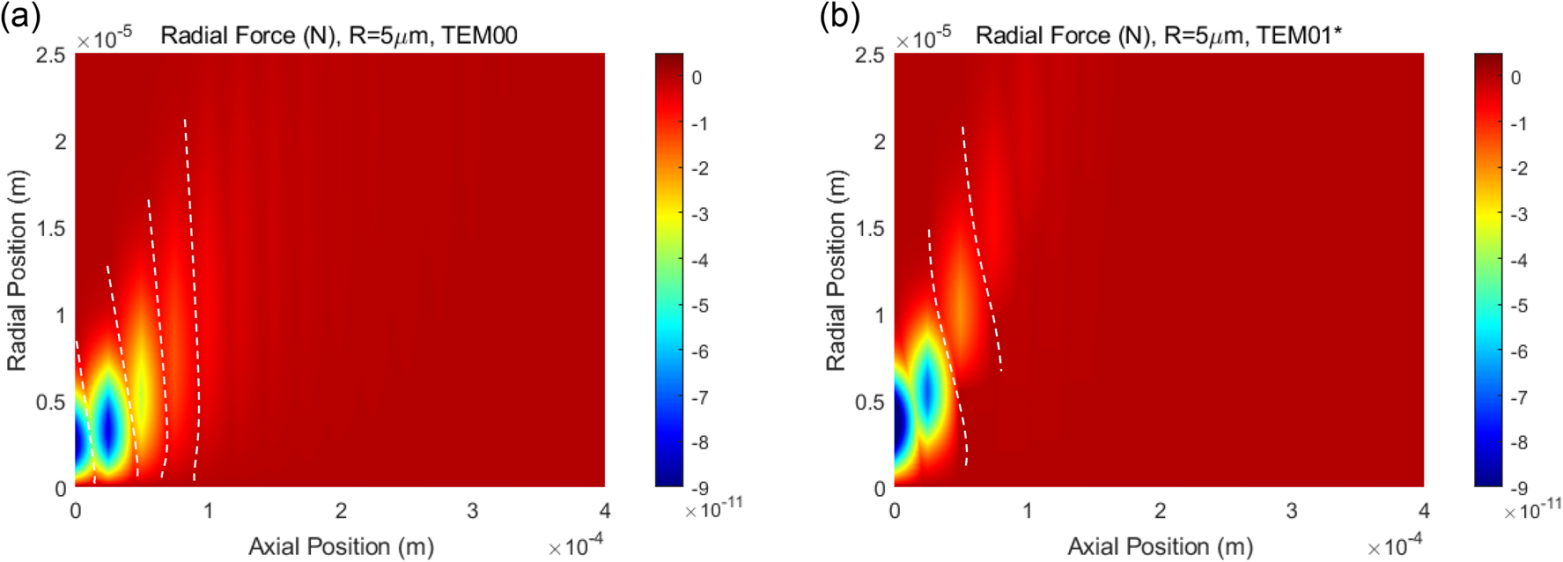
Optical radial force map exerted on HMM particle for (**a**) *TEM*_00_ and (**b**) $TEM_{01}^{*}$ laser beam incidences. Dashed lines in the figure indicates “Zero-force” paths, at which radial stabilization of HMM particle exists.

Exerted optical radial forces on the studied HMM particle are also mapped in spatial domain for *TEM*_00_ and $TEM_{01}^{*}$ laser beam incidences, see Fig. 7a, b respectively. Examining the radial force maps in detail, “Zero-force” paths exist at certain intervals, which are indicated by dashed lines in the figure. Zero-force paths evidence the radial stabilization of HMM particle that could be provided even at off-axis positions of the laser beam-center. The superiority of the proposed HMM particle is that zero-force paths are apparent for both *TEM*_00_ and $TEM_{01}^{*}$ laser beam incidences, which is not possible for only dielectric/only metal particle cases.

## Conclusion

Optical levitation and trapping of transparent micro-spherical Type-I HMM particle that consists of alternating layers of Ge-AZO has been investigated for the first time in the literature in this work and thought-provoking analytical results are obtained. We first derived the power reflectance and transmittance in the air-HMM interface, and utilized the analytical derivations for upgrading the conventional axial and radial force equations. The derived axial/radial force equations are compatible with HMM particle case under optical radiation pressure and corresponding forces are calculated accordingly. Then, in the ray optics regime, the optical levitation trap simulations are performed and the results are compared with the corresponding dielectric particle case. The main achievement in this study is that the radiation force boosted thanks to the negative refraction effect, achieving more than ~ 8 times force enhancement in the axial direction for the HMM case compared to the corresponding dielectric particle case. The physical interpretation of the enhancement in the optical force has been analyzed and explained by the Minkowski’s momentum relation. The second observation is The HMM particle provides a better stabilization and controllability than the conventional dielectric particle case. Optical radial force is also mapped in spatial domain for *TEM*_00_ and $TEM_{01}^{*}$ laser beam incidences, exhibiting zero-force paths at certain intervals, which is another superiority of the proposed system. We believe that the numerical results obtained in this study are just a small beginning of a new field of research especially in the quantum computing area owing to the inherent stabilization property of HMM particles under laser beam radiation. Thus, we encourage the experimentalist readership to perform and validate the outcomes of this study experimentally.

### Supplementary Information

Supplementary Figures.

## Data Availability

The datasets used and/or analysed during the current study available from the corresponding author on reasonable request.
