# Supplementary material for: Enhancement of optical levitation with hyperbolic metamaterials
Source: Sci Rep. 2024 Jan 19;14:1734. doi: 10.1038/s41598-024-51284-4 (PMC10799002; doi:10.1038/s41598-024-51284-4)
Supplement: Supplementary file 1 — Supplementary Figures. [file 41598_2024_51284_MOESM1_ESM.docx]

**Supplementary Information for “ENHANCEMENT OF OPTICAL LEVITATION WITH HYPERBOLIC METAMATERIALS”**

**Ufuk Paralı^*1^, Kadir Üstün^1^, İbrahim Halil Giden^1,2^**

*^1^ASELSAN Inc., Mehmet Akif Ersoy Mah. İstiklal Marşı Cad. No:16, 06200 Yenimahalle-Ankara,Türkiye*

*^2^ Gazi University, Faculty of Engineering, Department of Electrical and Electronics Engineering, 06570 Ankara, Türkiye*

*^[*](mailto:*uparali@aselsan.com.tr)^[uparali@aselsan.com.tr](mailto:*uparali@aselsan.com.tr)*

| 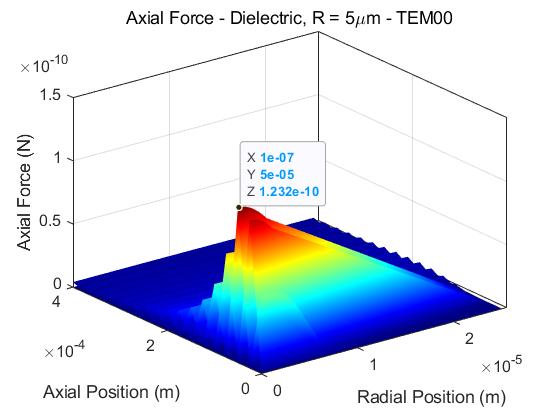 | 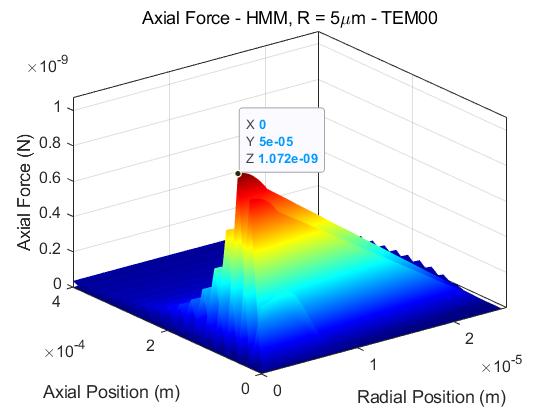 |
| --- | --- |
| 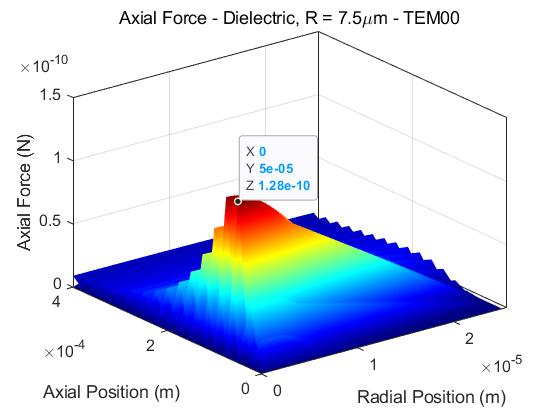 | 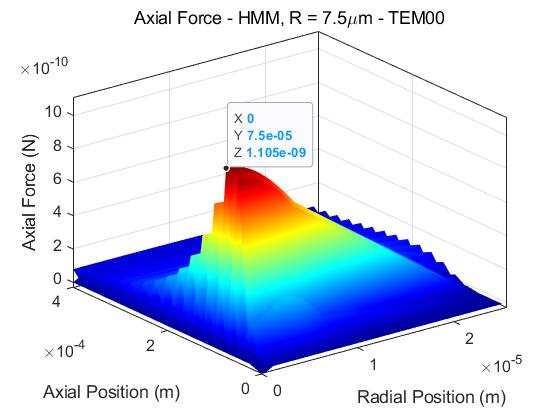 |
| 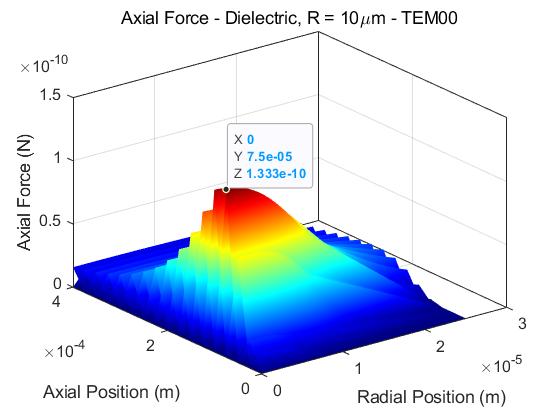 | 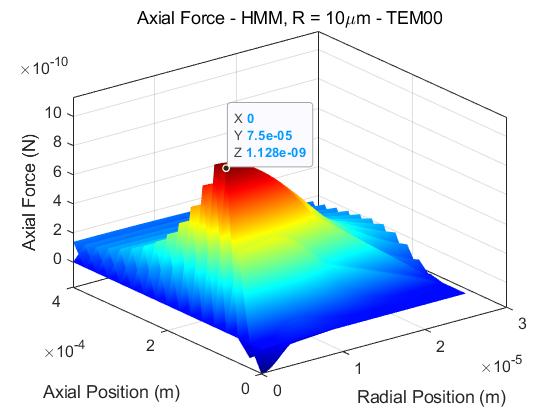 |
| 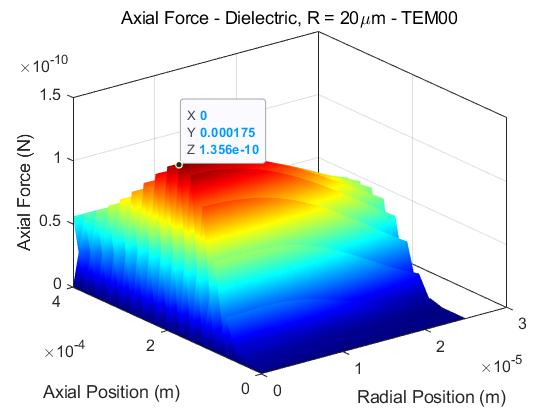 | 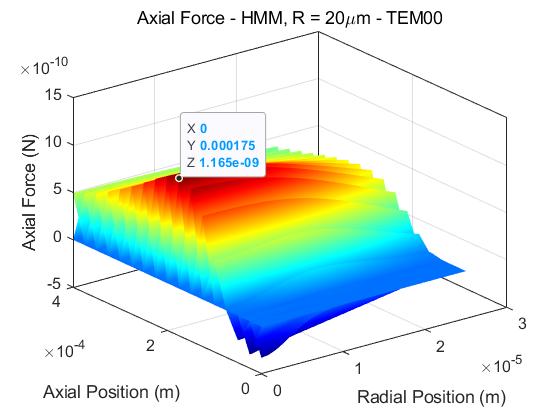 |

**Fig. S1.** Optical force profiles of dielectric/HMM particles in the axial direction for varying particle sizes.

| 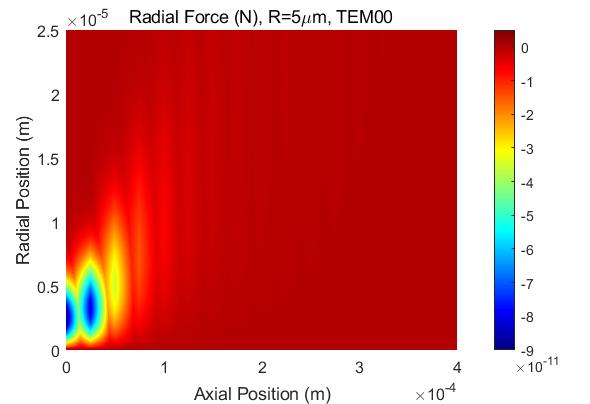 | 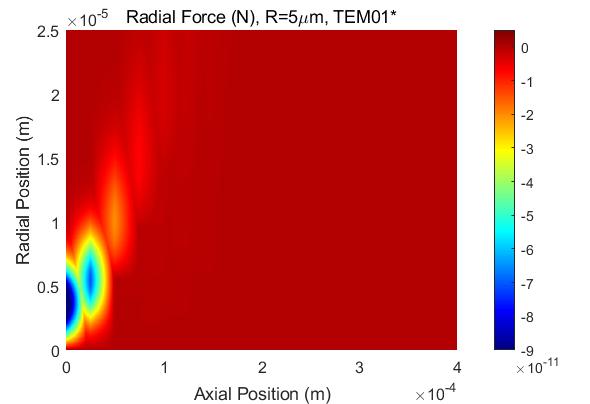 |
| --- | --- |
| 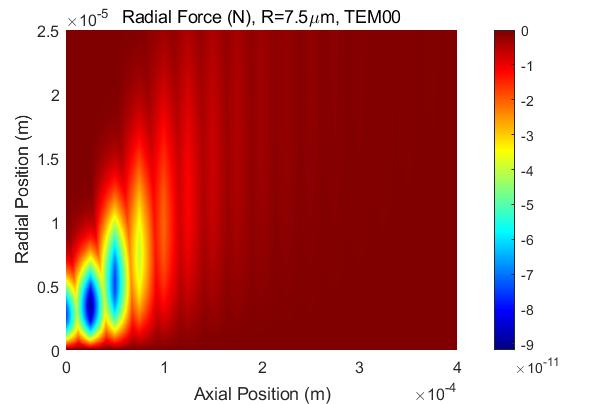 | 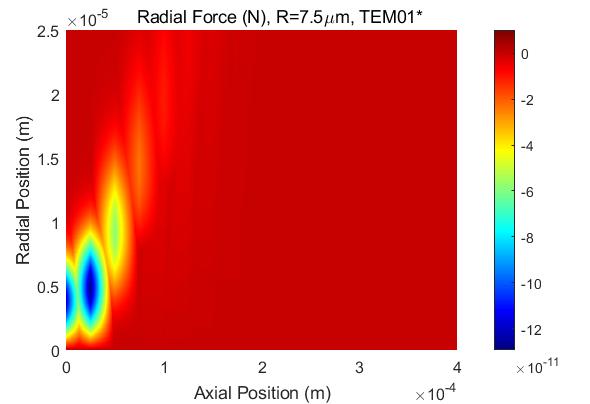 |
| 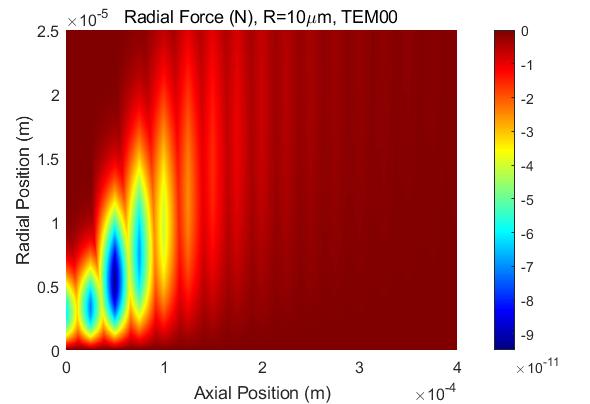 | 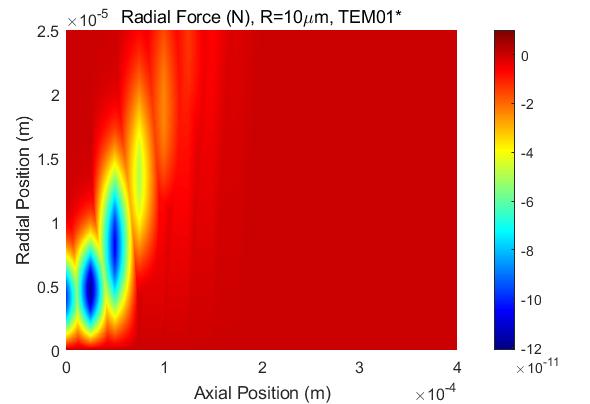 |
| 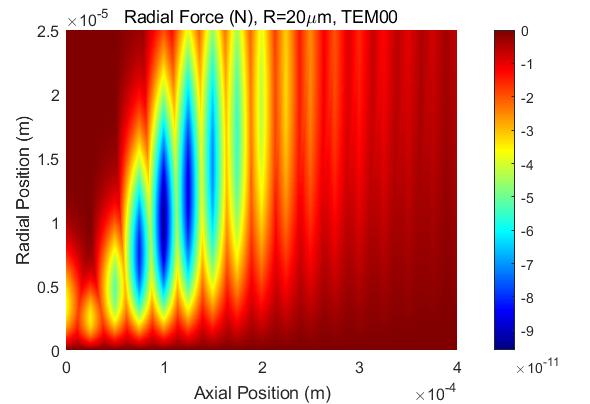 | 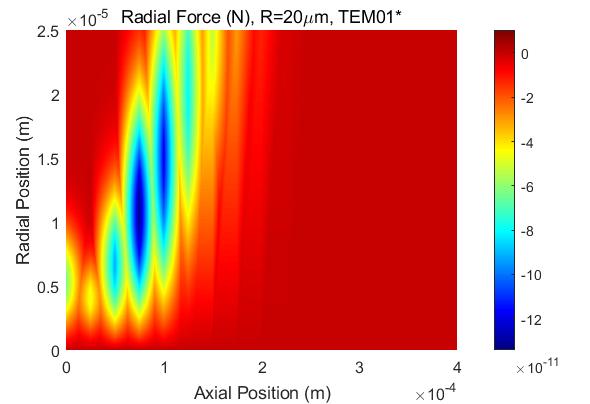 |

**Fig. S2.** Optical radial force map exerted on HMM particle for ${TEM}_{00}$ and ${TEM}_{01}^{*}$ laser beam incidences depending on varying particle dimensions.

| 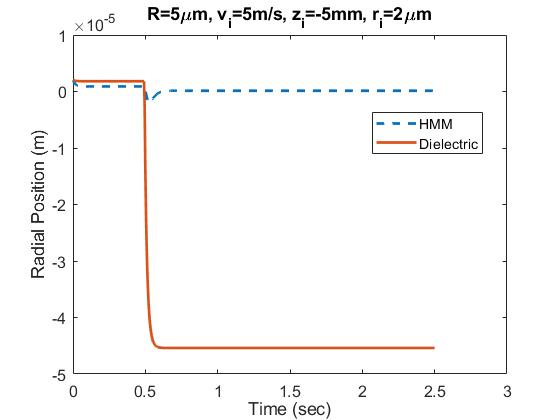 | 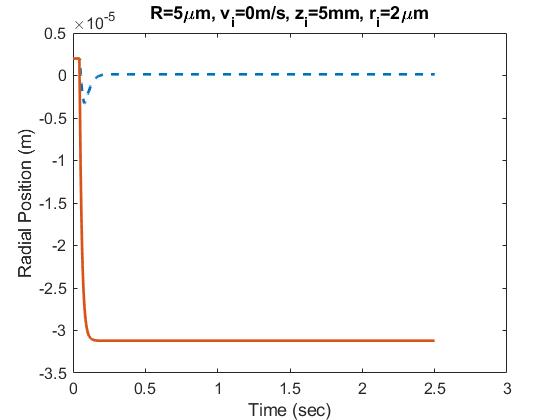 |
| --- | --- |
| 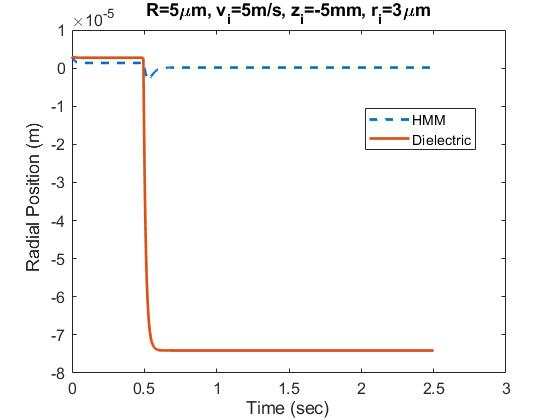 | 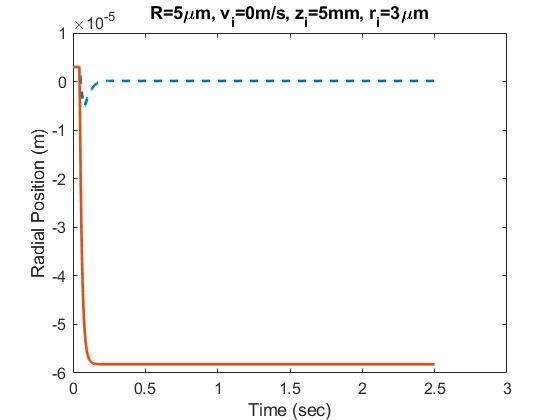 |
| 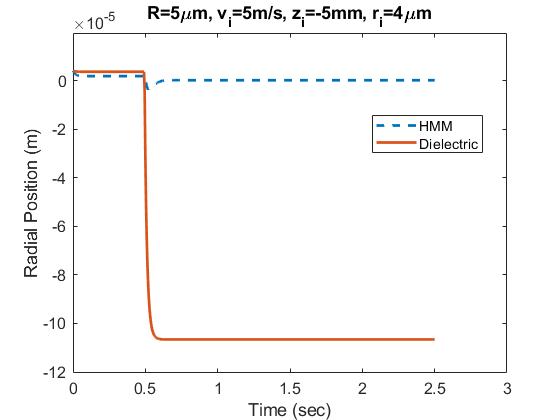 | 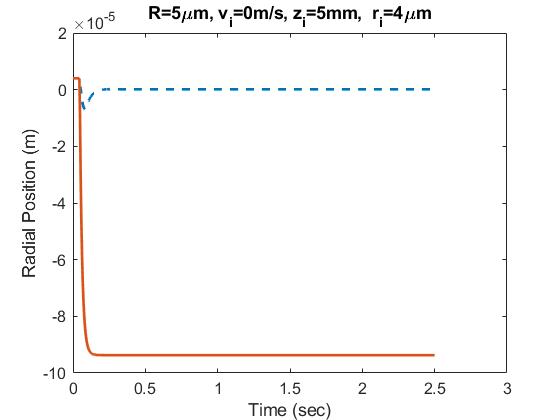 |

**Fig. S3.** Radial stabilization performance of transparent micro-spherical Type-I HMM and dielectric particles for varying input parameters.
